# Combinatorial models for evolving representations of dynamical behaviours in biological networks

**DOI:** 10.1098/rsfs.2025.0029

**Published:** 2025-08-22

**Authors:** Madalena Chaves, Elizabeth Remy, Elena Queirolo

**Affiliations:** ^1^ Université Côte d’Azur, Inria, INRAE, CNRS, Team MACBES, France; ^2^ Aix Marseille Université, CNRS, I2M, Marseille, France; ^3^ Université de Rennes, IRMAR UMR 6625, 35000, Rennes, France

**Keywords:** combinatorial models, Boolean models, switching systems, piecewise linear models, dynamical behaviour, biological networks, genetic regulatory networks, network inference

## Abstract

This theme issue is dedicated to ‘combinatorial’ models and their capacity to represent biological regulatory networks. As opposed to standard models using differential equations, ‘combinatorial’ models constitute a class of systems whose main property is that their global dynamics can be efficiently computed and their characterization rigorously proved, even in high-dimensional systems. Combinatorial models include Boolean networks and other discrete formalisms, piecewise affine or threshold networks and switching system models, as well as ‘coloured’ networks. This issue includes several recent examples of these formalisms and their contribution to analysis of biological networks.

Combinatorial systems typically use one or more of the following components: discrete variables, discrete time, discretized state space or discretized parameter space. Through these components, rigorous descriptions can be provided for the solutions and dynamical behaviours of the systems. Such discrete approaches provide elements to systematize large amounts of biological data, cluster regions of parameters that correspond to the same qualitative dynamics and propose models for high-dimensional biological systems.

Each of the above-cited formalisms has been widely studied by itself, but another interesting problem is to identify the various connections between them and explore the hierarchies and possible exchanges among formalisms, to obtain more complete and efficient biological representations. For instance, some well-known links have been characterized between Boolean or multi-valued Boolean networks and switching networks. Can these links be further generalized to include a more complete classification of all the possible dynamics of a given system? By their construction, these formalisms are naturally open to the composition of large networks by the interconnection of smaller networks or, conversely, to the decomposition of large networks into smaller modules to allow for a more detailed analysis.

To propel and stimulate further research on combinatorial networks, we need new strategies and tools that will help us to deal with and overcome the numerical and theoretical challenges often arising from an increasing complexity due to increasing network dimensions. This theme issue gathers a collection of articles, including a tutorial, a review and three original research papers, to help us through the use and analysis of combinatorial systems for modelling in the biological sciences.

Boolean networks and other logical models have long been used to represent and analyse the dynamics of biological regulatory networks. During the last four decades, a large number of software tools have been developed by different groups, some of which recently founded CoLoMoTo (Consortium for Logical Models and Tools) [1], a software suite that includes several tools for logical models and uses the standard SBML format. In a detailed tutorial, Noël *et al.* present tools for visualization of the network, attractor analysis and computation, but also stochastic simulation of trajectories associated with node transition rates, illustrated by a running example on mammalian cell proliferation.

Another class of widely used combinatorial models are piecewise affine or switching systems. These may be viewed as ‘hybrid’ models since they are defined through a family of ordinary differential equations with linear degradation rates and piecewise constant production rates. There are also a number of threshold parameters for each variable, which partition the state space into hyper-rectangles or domains. The systems’ trajectories may then be viewed as a transition between domains. In their review article, Perkins *et al.* recapitulate this class of systems with a focus on the inverse problem of determining the underlying regulatory network interactions based on the observed dynamics. Four different inference algorithms are reviewed, based either on sequences of maxima and minima or on sequences of logical states found by discretizing the dynamics.

A class of piecewise affine systems is also used by Böbel *et al.* to study the dynamics of the mammalian circadian clock with a protein sequestration term. In this modelling framework, the authors are able to show the existence of a periodic orbit for the system, over a large region of parameters. In the second step, the authors analyse the phase response curve of the periodic system, showing that it admits a very specific form, which implies that external perturbations can affect the system only in a narrow window of the periodic orbit.

The problem of regulatory network inference is again approached by Klomp *et al.* here studied through the lens of artificial intelligence. The starting point is an incomplete regulatory network, which is then studied with state-of-the-art machine learning techniques based around a graph autoencoder to retrieve the missing edge. Further analysis is then necessary to distinguish correctly added edges and spurious ones.

Finally, a connection between monotone Boolean networks and Petri nets is studied by Andreas *et al,* who establish bridges between monotone Boolean models, finite multilevel discrete systems and ordinary differential equation switching systems for regulatory networks. The authors propose a unified approach based on expanded networks and Petri net frameworks. It enables the characterization of all attractors and the global structure of dynamics, providing a complete description of the qualitative behaviour of complex biological systems across parameter space. This combinatorial methodology overcomes classical computational limitations by avoiding exhaustive state space exploration while preserving the ability to identify robust dynamical features of gene regulatory networks.

This theme issue is motivated in part by a recent one week workshop organized at the Lorentz Center (University of Leiden), with the theme of computing combinatorial dynamics in high-dimensional biological networks [2]. The aim of this workshop was to gather researchers currently working with different forms of combinatorial models. As an outcome of this workshop, several topics have emerged, some of which are now included in this theme issue.

## Data Availability

This article has no additional data.
